# Surgical Patching in Congenital Heart Disease: The Role of Imaging and Modelling

**DOI:** 10.3390/life13122295

**Published:** 2023-12-02

**Authors:** Yousef Aljassam, Massimo Caputo, Giovanni Biglino

**Affiliations:** 1 Translational Health Sciences, Bristol Medical School, University of Bristol, Bristol BS2 8HW, UK; ce21129@bristol.ac.uk; 2 Bristol Heart Institute, Bristol Medical School, University of Bristol, Bristol BS2 8HW, UK; m.caputo@bristol.ac.uk; 3 Cardiac Surgery, University Hospitals Bristol & Weston, NHS Foundation Trust, Bristol BS2 8HW, UK

**Keywords:** patches, modelling, imaging, design, 3D printing, patient-specific, congenital heart disease

## Abstract

In congenital heart disease, patches are not tailored to patient-specific anatomies, leading to shape mismatch with likely functional implications. The design of patches through imaging and modelling may be beneficial, as it could improve clinical outcomes and reduce the costs associated with redo procedures. Whilst attention has been paid to the material of the patches used in congenital surgery, this review outlines the current knowledge on this subject and isolated experimental work that uses modelling and imaging-derived information (including 3D printing) to inform the design of the surgical patch.

## 1. Introduction

### Cardiac Patch Materials and Patch Design

Congenital heart defects (CHD) are considered the most common cause of mortality amongst infants [1,2]. The incidence rate can be as high as 7.5% of all live births if small lesions are included [3]. CHDs are a diverse group of structural anomalies affecting the heart and the cardiovascular system [4].

Surgical patches are used extensively to repair several CHDs, employing either a synthetic patch (e.g., Dacron) or natural tissue (e.g., pericardium) [5]. However, current surgical practice is still not optimised, and many studies have reported complications in long-term outcomes [6,7,8]. Previous reviews have focused on patch materials, including Pok and Jakot [5], Zhang et al. [9], Liu and Qui [10], and Pievandi et al. [11]. The material used for the construction of patches is an important topic to consider, as the currently used materials have several issues regarding deterioration and calcification as well as the potential for growth. For example, the most recent (2023) review on patch materials by Pievendi et al. discussed the different materials used in the treatment of congenital heart disease such as autologous pericardium, which is considered the golden standard but presents important limitations such as insufficient quantity and the use of glutaraldehyde to fix the pericardial tissue itself [11]. Fixation was considered as a routine procedure, but it has been shown to negatively affect the durability of the tissue [12]. On the other hand, to abandon the use of glutaraldehyde would make the tissue more difficult to manage in surgery. Other biological materials have been extensively used for the purpose of creating suitable patches, such as bovine and porcine pericardium, and although they are not disadvantaged by formation of thromboembolisms as in the case of synthetic patches, they still calcify, degenerate over time, and lack tissue growth, which could lead to reoperation [11]. The use of homografts on the other hand may have lower reoperation rates, but they may still cause obstruction and stenosis, especially in paediatric patients [13]. The use of the Contegra conduit, which is made from bovine jugular veins and is used for right ventricular outflow tract reconstruction, may be a better substitute to homografts in regard to the risk of stenosis, but studies show higher reoperation rates [14]. There is ongoing research on tissue engineering to develop more optimised patch materials with a potential for growth, such as the use of composite polymers, e.g., chitosan and fibrin, for patching heart muscle [15]. As indicated, the topic of patch materials has been more extensively researched and discussed, whilst less attention has been devoted to the methods that can inform and potentially optimise the design of patches, which is why we decided to focus this review on this specific topic. The goal of this review is to map current knowledge on and discuss the importance and potential of modelling in guiding the design of patches. Advancements in technology and medicine combined with the fact that even successful repair does not result in complete freedom of complications and reintervention have led to the pursuit of tailored (patient-specific) patch designs and personalising techniques of surgical repair. This narrative review presents these recent approaches discussing how they may inform innovative patch designs and thus potentially improve clinical outcomes.

## 2. Methods

We performed a literature search focusing on surgical patch design. Different databases were searched to map all the relevant literature, namely Pubmed, Cochrane, Scopus, and Google Scholar. The keywords searched included combinations of patch AND modelling OR computational fluid dynamics/CFD OR imaging OR design OR 3D printing OR simulation OR pre-surgical planning OR patient-specific, limiting searches to the field of congenital heart disease. Studies pertaining to patch design through use of imaging, 3D printing, and modelling in the context of congenital heart disease were thus included in the paper. Studies that discuss surgical patch techniques only, without the aid of imaging and/or modelling, or studies that were unrelated to congenital heart disease were excluded.

## 3. Results

As summarised in Table 1, 13 studies were found in the above literature search. These were focused on patching the aorta, pulmonary arteries, right ventricle, and atrial septal defects. These studies were published between 2015 and 2022, a timeline that seems in line with advances in imaging and modelling. The findings are discussed according to the location of the patch.

### 3.1. Aorta

**Figure 1:**
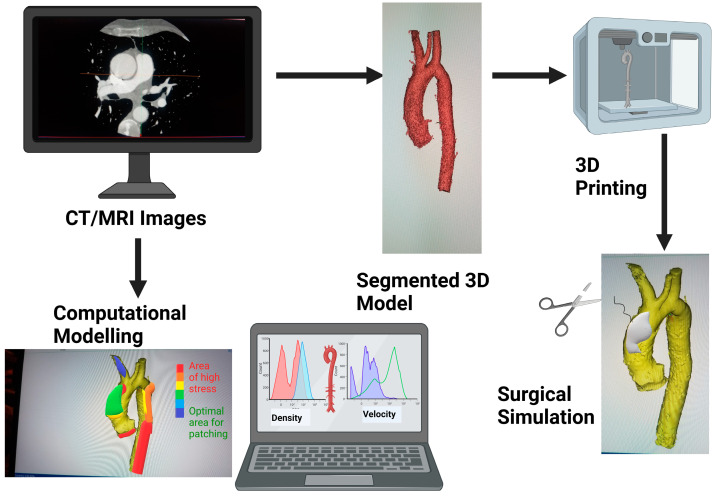
Schematic showing how medical images can be 3D printed or be used for modelling for surgical simulation. Graphics created with Biorender.com.

Both Chen et al. [16] and Belitsis et al. [17] sought to use 3D printing (Figure 1) to simulate the Norwood procedure and design an aortic patch for hypoplastic left heart syndrome (HLHS) patients. Chen and colleagues 3D printed pre- and postoperative models of the aortic arch and ascending aorta, and editing applications were used to edit the postoperative images and revert the anatomy to a preoperative state using data from preoperative ultrasound imaging. Next, they simulated the aortic reconstruction procedure digitally in order to create optimised patches that had a more compatible shape and size according to the patient’s anatomy. The models and patches were then 3D printed so that they could be used for surgical simulation, helping the cardiac surgeons ensure that the newly designed patches are feasible. 

Belitsis et al. have 3D-printed models of ductal arches, which were used to take measurements informing the length, thickness, and curvature of a purposely designed patch. The choice of ductal arches was made because the ductal arch has an identical geometry to the aortic arch in terms of curvature and height and thus serves as a guide for designing a patch for the aortic arch.

**Figure 2:**
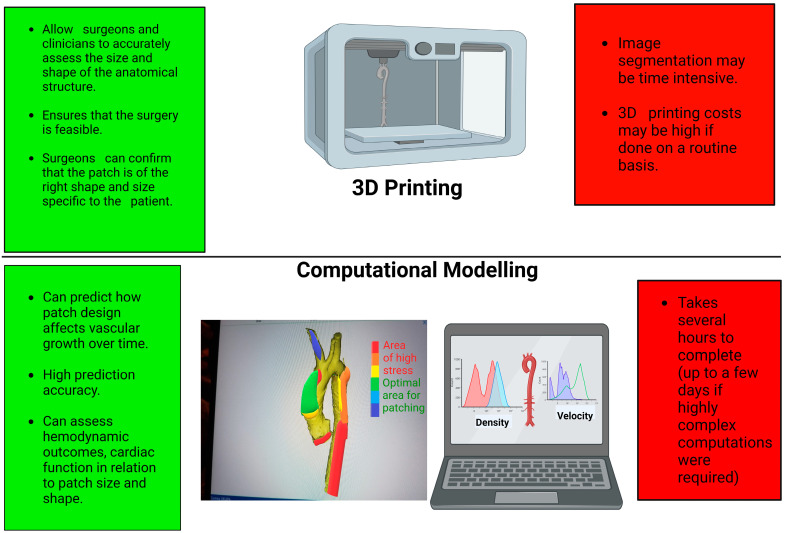
Schematic showing the advantages (in green) and disadvantages (in red) of 3D printing and computational modelling. Graphics created with Biorender.com.

Mayoral et al. [26] also used 3D printing to guide the design of patches, in this case for a paediatric patient with hypoplasia of the aortic arch. Their approach to create an ideal patch, both in terms of design and material, also involved computational fluid dynamics (CFD, Figure 2) and tissue engineering in order to design a patient-specific autologous cell-seeded patch for the aorta. They used this method to design two patches based on the incision length made during surgical simulation and were validated by performing the surgery on 3D-printed models replicating the same anatomies. Next, they used CFD to analyse which patch yielded the best haemodynamic outcome, defined as the configuration yielding lowest wall shear stress and pressure. The quantitative information from CFD can be useful not only to assess how changes in the shape and size of the patch can be refined but also the haemodynamic implications of different designs.

### 3.2. Pulmonary Arteries

Lashkarinia and co-workers [18] simulated the surgery of tetralogy of Fallot, specifically patch augmentation of the main pulmonary artery, using a finite elements model. They assessed the mechanical and haemodynamic performance parameters of patch repair in order to optimise the patch design. The accuracy of the prediction model was validated experimentally, and it was shown to have approximately 97% accuracy, suggesting its feasibility in clinical practice. The parameters assessed were the number of incisions made during the surgery to patch the artery as well as their length and shape. The thickness of the patch was another parameter assessed in this study and deemed important because it could affect the shape of the patched artery in addition to the postoperative performance. Increasing the patch thickness resulted in decreased deformation and stress. This study also remarked on the stress applied to the artery because of the surgical incision. The longer the incision, the higher the stress, and a greater stenosed area will also result in an increased stress. However, a shorter incision length (<16 mm) also resulted in regions of increased stress along the patched pulmonary artery. The length of the incision and the resulting stress were thus shown to have an effect on residual stenosis. This study concluded that using a PFTE material patch with a longer surgical incision seemed to be the most ideal method to reduce stresses and that thicker patches may be preferable to thinner ones. The thickness of the patch seemed to play a more substantial role, from a mechanical standpoint, than patch material. More distensible patches were shown to be preferable but still avoiding a stiffness lower than the native tissue.

Another advantage of computational modelling is that it can also help simulate long-term outcomes of patch augmentation. The same group [19] used another simulation to predict the long-term outcomes of patch augmentation of the pulmonary arteries; in particular, they looked into how the presence of the patch affects the growth of the arteries up to 17 years after surgery. Simulations showed the proximal growth of the vessel and increased stiffness at the corners of the patch as well as at the original stenotic region. Using such computational methods and simulations, surgeons may be able to use a patient-specific patch i.e., with optimised shape and material properties, which in turn can potentially lead to better clinical outcomes. Thus, both studies show that the application of computational modelling is especially useful for surgeons in determining vascular changes caused by patching (patch shape and incision length) both during and long after the procedure through prediction. This is a key advantage since patch design is not a one-size-fits-all, and, no matter the level of experience, the surgeon cannot accurately predict the long-term outcomes of patch augmentation for a specific patient. However, it is important to note that many clinical parameters need to be simplified in order to carry out the simulation, which will not fully represent the actual physiological profile of the patient. Because of this, the computational model will not necessarily be able to predict outcomes accurately, and validation of this method is complex, particularly in vivo [29].

Similarly, Miyaji et al. [23] also used CFD to determine the optimal anatomy of the pulmonary arteries with the most efficient haemodynamics. This was achieved by reconstructing preoperative computed tomography (CT) images and then editing the models to have a postoperative anatomy by enlarging the vessel diameter as a way of simulating patch augmentation. Hemodynamic analysis was performed, and the model with the least energy loss and wall shear stress was identified. The patch was designed by measuring the preoperative geometry and the edited postoperative model and calculating the difference. Multiple cross-sectional measurements of the width of the patch were taken to establish the patch size and shape. This study compared the results of the simulated surgery with the actual surgery, which provides better validation data. It is important to note that several variables can affect simulations (their running time and computational cost as well as their accuracy), including for example the choice of flow regime (steady vs. pulsatile) and the material properties of the blood vessels and of the patch. Furthermore, to obtain more detailed haemodynamic information, clinicians should use magnetic resonance imaging (MRI) with 4D flow and inform in great detail the flow boundary conditions of the simulations.

### 3.3. Right Ventricle

Tang and associates [20] used a combination of MRI and computational modelling to optimise the patch used for the right ventricle, specifically for patients undergoing pulmonary valve replacement. They created several models of the right and left ventricles with two differently sized patches from segmented MR images. Cardiac pressure and right ventricular volume were considered when creating these models. One model had a conventionally sized patch with minimum trimming of the right ventricle scar tissue, while the other model had a smaller patch with more aggressive trimming of the right ventricle scar tissue. They reported lower stress and strain by up to 50% when the small patch was used compared to the conventional patch. Designing a patch with less stress implies that the ventricle will not be subjected to higher stress, which has functional implications in terms of cardiac work and energetics. Further refinements of the simulation can improve the analysis, such as including the simulation of valve mechanics.

Miyazaki et al. [21] used an unconventionally shaped patch for right ventricular outflow tract (RVOT) reconstruction. The patch was made from expanded polytetrafluoroethylene (PTFE) and was valve-shaped with a fan-like appearance and had a bulging sinus that mimicked the actual shape of the native valve. The reasoning behind such shape is that it allowed greater opening of the valve and reduced energy loss. This unique design of the patch yielded good short and long-term outcomes in 157 patients as indicated by echocardiography and MRI angiography. The reported outcomes in terms of pulmonary regurgitation after a followup of >50 months were mild in more than 50% of patients and severe in about 1% of patients. In terms of RVOT stenosis, it was mild in more than 90% of patients and severe only in less than 2%. About 6% of patients required reintervention, with 95% survival at 15 years. However, they did not compare these patches with conventional patches in their study to see whether their patch offered better outcomes. To prevent pulmonary regurgitation in these patients, it is recommended that valved patches augment the area of greater curvature where the flow is highest. CFD assessment could be combined with this patch design to make it more patient-specific, with optimal haemodynamic outcomes.

The studies by Liang et al. [25], Ma et al. [27], and Mendez et al. [28] all used 3D heart reconstructions from CT images, which were 3D printed to plan and simulate the surgical procedure for patients with ventricular septal defects (VSD). The VSD patches were designed by taking geometric measurements from the 3D models. The 3D models and the 3D-printed patches were used to guide the surgical procedure and aid in making and trimming the patches. The virtual and 3D-printed models improved diagnosis, as the number of VSDs were not previously known, and they were considered superior to conventional diagnosis while also informing about the optimal patch size and shape. Again, these studies highlight the importance of 3D printing in surgical planning especially in cases of complex VSDs where the defects are difficult to visualize and assess two-dimensionally. In such complex cases, the surgeon may not be able to visualise the VSDs clearly, indicating the usefulness of the 3D-printed model in guiding the procedure and leading to its success.

Because of dose and use of contrast media as well as consistency, cardiac MR imaging may be preferred as an imaging source for the 3D reconstructions. However, if CT was to be used and the heart model needs to be 3D-printed then it is advised that triggering is used to ensure the heart is adequately filled with the contrast agent to generate an accurate high-density 3D model of the heart.

A paper by Giannopolous et al. [22] is a tutorial for applications for image segmentation and processing (Mimics and 3-matic, Materialise, Leuven, Belgium). This is worth mentioning for completeness because in their tutorial they show how to design a patch for a patient with double outlet right ventricle and VSD. This was achieved by virtually drawing the patch directly onto the VSD in the 3D reconstructed model so that it matches the patient’s anatomy in the 3-matic software (which can also be used to design tailor-made patches, implants, and prostheses). It would be helpful to produce more tutorials in other types of CHD using various modelling techniques and/or packages to serve as guidelines for clinical practice.

### 3.4. Atrial Septal Defects

Nakamura et al. [24] designed two different patient-specific patches for correcting the atrial septal defect in a paediatric patient during an intra-atrial rerouting procedure, which was simulated via a 3D-printed model of the heart. The patches were created using surface modelling and were slightly different in size with a small difference of the shape at the anterior border of the atrial defect. Both patches were feasible in the surgical simulation performed by the cardiac surgeon. However, the CFD showed that for one patch the blood flow of the superior vena cava into the right atrium was more abnormal, with double energy loss compared to the other patch design. This demonstrates that the size and shape of patches for any CHD may potentially have some effect, in specific anatomies, on haemodynamic/functional variables and therefore potentially on clinical outcomes in the long term. It also shows that surgeons may create different patch designs depending on their expertise and preference. Surgeons typically design the patch intraoperatively under cardiac arrest according to what they see, but the cardiovascular structures appear differently with blood flow restricted than when blood flow is resumed. This change in structure, no matter how slight, will affect different patch designs that may not adequately match the anatomy of interest, in turn potentially leading to future complications and adverse haemodynamic effects or even clinical outcomes.

Results from some of these modelling-based studies suggest that patch size, especially in transannular patching, should be kept as small as possible since large patch sizes were shown to increase stress and strain. From a materials standpoint, patch material properties and thickness should also be considered. Compared to current materials (e.g., PFTE or bovine pericardium) that present issues including the risk of calcification over time, new materials are being developed particularly exploring materials compatible with 3D-printing technology, thus allowing the implementation of the abovementioned personalisation approaches [16]. Such materials should match the mechanical properties of native tissue, and it should be acknowledged that, with the rapid advancements in rapid prototyping, more materials are increasingly becoming compatible with the technology.

## 4. Discussion

In recent years, researchers have begun to appreciate the potential of personalised patch design as evidenced by the majority of the discussed articles (all published within the last five years). This is informed by clinical observations: clinical studies have indicated that in many cases patch augmentation results in abnormal anatomical configurations, with long-term implications on haemodynamics and clinical outcomes despite a successful initial surgical repair [16,30].

From a clinical standpoint, different options are available to the surgeon, and clinical practice reflects these options. For paediatric patients, reconstruction of the aorta is usually performed using pulmonary homograft patches, as it has been reported that using such patches have more favourable outcomes compared to bovine pericardium, especially in Norwood patients [31]. A study by Lewis et al. reported that the 15-year survival of pulmonary homograft for aortic arch reconstruction in 124 patients was over 80%, while freedom from intervention was close to 90% with no signs of calcification or degeneration [32]. On the other hand, in addition to autografts and homografts, bovine or synthetic patches may be used for adults or for pulmonary artery reconstructions since they come with their own advantages such as lower cost, availability, mechanical strength/durability, resistance to degeneration, and ease of handling [33,34].

Patch design has a significant impact on the repair of the defect, including the haemodynamics of the blood vessel, and contributes to long-term outcomes [19]. Nonetheless, many surgeons still subjectively design the patch intraoperatively based on previous experience and intraoperative assessment. As mentioned, designing a patch based on intraoperative visual assessment may imply not optimising the patch design to the actual pressurised configuration [24]. Once blood flow is reinstated, the structure of the vessel will change potentially creating a patch–vessel mismatch that increases haemodynamic stress on the tissue.

The current methods for quantitative assessment of patch design mainly include (a) CFD to evaluate the haemodynamics of the blood vessels with different patch designs and predict cardiac function and (b) 3D printing to manufacture models of the vessels or heart and simulate the surgical procedure and assess patch augmentation feasibility. Surgeons can work with other health professionals as well as engineers in implementing these techniques in clinical practice as part of the surgical planning. Whilst the scenarios discussed in this review are limited reflecting the limited number of studies that are beginning to implement such a personalised approach, other CHD scenarios could also in principle benefit from it. Importantly, the use of technology including 3D technologies has been recently highlighted as a research priority in congenital heart disease, both paediatric and adult [35], and novel applications warrant exploration.

Finite element analysis (FEA) is a powerful computational method that can be applied to the assessment of material properties and design [36]. Thus, it can be used to model and study the effects of a patch on the native tissue including yielding important mechanical information (e.g., stress and strain). For example, one of the discussed studies used FEA software to specifically assess the effects of patch properties on vascular remodelling [19]. In this study, FEBio software was utilised, providing a rich library of plugins that can perform various simulations [37]. The application of FEA and using software such as FEBio can measure the mechanics, hyperelasticity and viscoelasticity of materials and how tissues interact with said materials in terms of growth and remodelling [38]. Interestingly, these simulations can also be valuable in providing insights into the biochemical interactions between two materials, such as patch material and native tissue. This is especially useful when assessing novel biomaterials or tissue-engineered patches, whose properties could be measured and input into simulations. Other software such as Simvascular is more specialised in cardiac remodelling and offers the capability of simulating haemodynamics providing complex and accurate fluid–structure interactions, taking into account wall properties such as degree of elasticity and thickness [39]. Fluid structure interaction (or FSI) simulations are very important in this context to account for material properties of the patch. Experimental validation of simulations remains of paramount importance to demonstrate the accuracy of the computational vis-à-vis the complexity of the simulations (likely time-intensive simulations requiring high computational power). Proving information on computational results’ reliability and quantifying statistical variability of virtual surgery simulations, such as including confidence intervals [40], is also important and discussed in the modelling literature.

Another imaging-based computational approach that could be relevant in this context is the implementation of statistical shape modelling, particularly using a longitudinal atlasing methodology [41], to study morphological variations of patched vessels over time and thus provide information on variability in shape to inform new designs.

From a 3D-printing standpoint, the manufacturing of models for surgical practice is advancing [42], as is the knowledge of materials and biofabrication techniques to 3D print cardiac patches. The feasibility of printing vascularized patches according to the patient’s anatomy has been demonstrated [43], studying their structure and function, and more research in this area is welcome to advance and consolidate the use of 3D technologies to support congenital cardiovascular surgery.

## 5. Conclusions

In conclusion, imaging-based techniques such as 3D printing and computational simulations can contribute to advancing research in novel surgical patching. The use of 3D printed models of congenital heart defects can help surgeons to plan and simulate procedures for complex defects. Computational methods, such as CFD and FEA, can provide insights into the haemodynamic properties of blood vessels after patch augmentation, including virtual surgery simulations, contributing to identifying optimal design and size of the patch specific to a patient. The integration of such methods would promote a truly personalised approach to surgical patching in congenital heart disease.

## Figures and Tables

**Table 1:** Table of included papers. CT = computed tomography, FSI = fluid–structure interaction; HLHS = hypoplastic left heart syndrome; RVOT = right ventricular outflow tract. CAD = computer-aided design.

Author	Paper Title	Year	Method	CHD/Anatomy
Chen et al. [16]	Digital design and 3D printing of aortic arch reconstruction in HLHS for surgical simulation and training	2018	CT, 3-matic and Mehsmixer, 3D Printing, Surgical Simulation	HLHS/aorta
Belitsis et al. [17]	Ductal arch decoded: the use of its spatial fingerprint to design a Norwood type of patch	2022	3D Printing, Surgical Simulation	HLHS/aorta
Lashkarinia et al. [18]	Computational pre-surgical planning of arterial patch reconstruction: parametric limits and in vitro validation	2018	Finite Elements Model	Pulmonary artery
Lashkarinia et al. [19]	Computational modelling of vascular growth in patient-specific pulmonary arterial patch reconstructions	2021	Computational Fluid Dynamics	Pulmonary artery
Tang et al. [20]	Patient-specific MRI-based 3D FSI RV/LV/Patch models for pulmonary valve replacement surgery and patch optimization	2018	Fluid Structure Interactions	RVOT
Miyazaki et al. [21]	Use of an expanded polytetrafluoroethylene valved patch with a sinus in right ventricular outflow tract reconstruction	2019	Patch Design	RVOT
Nakumara et al. [22]	Patient-specific patch for an intra-atrial rerouting procedure developed through surgical simulation	2021	3D Printing, Surface Modelling, Surgical Simulation	Atrial septal defect
Miyaji et al. [23]	Novel surgical strategy for complicated pulmonary stenosis using haemodynamic analysis based on a virtual operation with numerical flow analysis	2019	Computational fluid dynamics, Virtual surgery	Pulmonary artery
Giannopolous et al. [24]	3D printed ventricular septal defect patch: a primer for the 2015 Radiological Society of North America (RSNA) hands-on course in 3D printing	2015	CAD, 3D Printing	Ventricular septal defect
Liang et al. [25]	Feasibility analyses of virtual models and 3D printing for surgical simulation of the double-outlet right ventricle	2022	3D Printing, Surgical Simulation	Ventricular septal defect
Mayoral et al. [26]	Tissue engineered in-vitro vascular patch fabrication using hybrid 3D printing and electrospinning	2022	3D Printing, Computational Fluid Dynamics	Aorta
Ma et al. [27]	Clinical application of three-dimensional reconstruction and rapid prototyping technology of multislice spiral computed tomography angiography for the repair of ventricular septal defect of tetralogy of Fallot	2015	3D Printing, Presurgical Planning	Ventricular septal defect
Mendez et al. [28]	Apical muscular ventricular septal defects: surgical strategy using three-dimensional printed model	2018	3D Printing, Presurgical Planning	Ventricular Septal Defects

## Data Availability

No new data was created.

## Abbreviations

CHD Congenital heart defects; HLHS hypoplastic left heart syndrome; VSD ventricular septal defects; ASD atrial septal defects; TOF tetralogy of Fallot; RVOT right ventricular outflow tract; CT computed tomography; MRI magnetic resonance imaging; PTFE Polytetrafluoroethylene.
